# Joint Genomic and Transcriptomic Analysis Reveals Candidate Genes Associated with Plumage Color Traits in Matahu Ducks

**DOI:** 10.3390/ani14213111

**Published:** 2024-10-29

**Authors:** Pengwei Ren, Liu Yang, Muhammad Zahoor Khan, Yadi Jing, Meixia Zhang, Chao Qi, Xin Zhang, Xiang Liu, Zhansheng Liu, Shuer Zhang, Mingxia Zhu

**Affiliations:** 1 College of Agriculture and Biology, Liaocheng University, Liaocheng 252000, China; 2 Shandong Animal Husbandry Station, Jinan 250010, China; 3 Jining Animal Husbandry and Veterinary Career Development Centre, Jining 272002, China

**Keywords:** Matahu duck, white plumage traits, genome, transcriptome, genetic markers

## Abstract

Plumage color is a key trait for identifying waterfowl breeds with significant economic importance. A white-feathered group has recently emerged within the native Matahu duck population, presenting an opportunity for breeding new lines. However, the genetic basis for this plumage variation is still unknown, necessitating further research. This study aims to identify the genetic mechanisms underlying the emergence of white-feathered individuals in the Matahu duck population through combined genome and transcriptome analysis, providing insights for selective breeding and the development of new white-feathered lines. In this study, a total of 1344 selected genes and 1406 significantly differentially expressed genes were identified through selection signal analysis and transcriptomic analysis, respectively. The functional enrichment of these genes revealed several key signaling pathways, including those related to cGMP-PKG, cAMP, PI3K-Akt, and MAPK. Furthermore, important candidate genes involved in melanin biosynthesis, such as *MITF*, *MC1R*, *TYR*, *TYRP1*, and *ABCB6*, were identified. Notably, 107 genes were detected by both methods, and, among these, *DGKI*, *GPRC5B*, *HMX1*, *STS*, *ADGRA1*, *PRKAR2B*, and *HOXB9* are suggested to play a role in melanin formation and potentially influence plumage traits. Through the integrative approach combining genomic selection signals and transcriptomic analyses, we identified several candidate genes directly associated with plumage color, including *MITF*, *TYR*, *TYRP1*, and *MC1R*, along with multiple signaling pathways linked to melanin formation. We hypothesize that the expression of *DGKI*, *GPRC5B*, *HMX1*, *STS*, *ADGRA1*, *PRKAR2B*, and *HOXB9*, detected by both methods, may be closely related to the regulation of plumage color traits. These findings provide a foundational basis for further research aimed at elucidating the genetic mechanisms governing plumage color variation in ducks.

## Simple Summary

Feather color has always been an extremely important economic trait in poultry production, as well as a complex, multifactorial biological trait. The Matahu ducks have been selectively bred to gradually form a new group with white plumage, which is very different from the plumage color of the original group. The molecular mechanisms underlying this unique trait remain incompletely resolved. In this study, genome selection signals and transcriptome sequencing results were jointly analyzed with the aim of finding key pathways and genes for duck plumage color formation. In this study, we searched for key genes and pathways associated with plumage color traits. These findings add a new experimental basis for the elaboration of the genetic mechanism of avian plumage color and provide effective theoretical support for the selection and conservation of local breeds.

## 1. Introduction

Coloration is one of the most recognizable characteristics of poultry, and understanding the mechanisms behind this coloration will enhance our knowledge of feather color formation. Furthermore, feather color is an economically and scientifically important trait in poultry breeding, serving as an indicator of breed quality [1]. White feathers are particularly valued for their economic advantages in down production [2], and regional preferences for feather color can influence meat quality perceptions [3], as seen with “three-yellow chickens” and black-feathered chickens in southern regions. Additionally, feather color may correlate with body weight, immune response, and reproductive traits, making it a key focus in poultry breeding [4].

Advances in molecular biotechnology have shed light on the genetic mechanisms underlying feather color, primarily driven by melanin synthesis and regulated by various genes [5]. Currently, more than 120 genes have been identified in animals that are involved in the determination of coat color, among which 40 loci controlling plumage color have been identified in poultry, such as Premelanosomal Protein (*PMEL17*), KIT Proto-Oncogene, Receptor Tyrosine Kinase (*KIT*), and Endothelin Receptor B2 (*EDNRB2*), etc., which play important roles in the formation, migration, and deposition of melanin [6,7,8,9]. Traditional studies have focused on single loci, but next-generation sequencing technologies have enabled more comprehensive analyses of genomics and transcriptomics, allowing for a broader understanding of feather color regulation [10,11]. For example, genome-wide association studies (GWASs) have identified candidate genes, such as microphthalmia-associated transcription factor (*MITF*) and melanocortin 1 receptor (*MC1R*), linked to feather color in ducks [1]. Transcriptome sequencing has also highlighted genes like tyrosinase related protein 1 (*TYRP1*), tyrosinase (*TYR*), and SRY-Box transcription factor 10 (*SOX10*), which play a role in melanin synthesis [3]. The genetic regulation of melanin-influenced plumage color traits is complex. The screening and confirmation of its primary effector genes, as well as gene-to-gene correlations, are still in the early stages of exploration, and the underlying genetic mechanisms remain worth investigating.

In this study, we utilized whole-genome resequencing technology to perform selective sweep analysis on both the hemp-feathered and white-feathered populations of Matahu ducks. By integrating these genomic data with transcriptome sequencing results, we identified differentially expressed genes shared across both populations, allowing us to pinpoint candidate genes involved in feather color traits. The findings of this study provide a genetic foundation for selecting and breeding white-feathered traits in Matahu ducks, offering valuable insights and technical guidance for future breeding efforts.

## 2. Materials and Methods

### 2.1. Test Animals and Sample Collection

**Figure 1:**
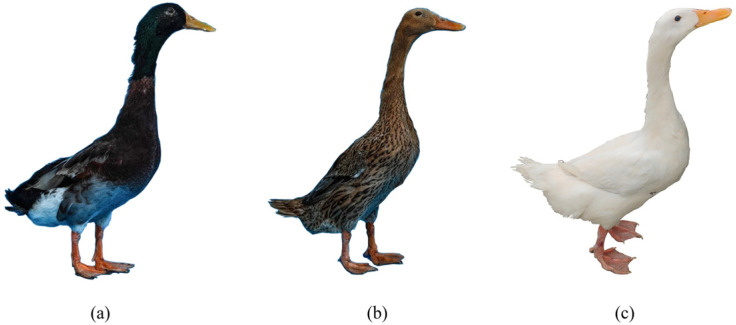
External characteristics of Matahu ducks: (**a**) male ducks of the original group; (**b**) female ducks of the original group; (**c**) white-feathered individuals (no distinction between males and females).

The original population of Matahu ducks and the newly hatched white-feathered population used in this experiment were collected from Xinhe Egg Duck Breeding Co., Weishan County, Jining City (Figure 1). Thirty ducks were selected from each of the original and white-feathered populations, for a total of 60 ducks. However, during the analysis, one outlier from the original population was excluded to ensure the independence and integrity of the data. Consequently, the final sample size was 59 ducks (29 ducks in the original population and 30 in the white-feathered population). These individuals were chosen based on similar physical characteristics, identical rearing conditions, and consistent incubation periods. Blood samples were collected from all individuals using the wing vein method for genomic DNA extraction. The blood samples were placed in EDTA anticoagulant tubes, transported at 4 °C, and subsequently stored at −20 °C for future analysis. The blood collection process was conducted over two separate time intervals. For further analysis, three male and three female ducks of the original group, along with three newly hatched white-feathered ducks (without sex differentiation), were randomly selected and humanely slaughtered according to the National Code of Conduct for the Care and Use of Experimental Animals. Skin tissues, including samples from the neck, back, and abdomen, were collected, immediately placed in RNAase-free freezing tubes, rapidly frozen in liquid nitrogen, and stored at −80 °C for further use.

### 2.2. Genome Resequencing and Variant Site Detection

After thawing the blood samples at room temperature, genomic DNA was extracted using the Tengen Blood Genomic DNA Extraction Kit. The integrity of the extracted DNA was assessed through 1% agarose gel electrophoresis, and DNA concentration was quantified using a spectrophotometer. The extracted DNA samples were sent to the Compass Agritechnology Co., Ltd. (Beijing, China), for library construction and high-throughput sequencing to generate raw data at a coverage depth of 30×.

The raw sequencing data were then subjected to quality control, where paired-end reads containing adapter sequences, excessive Ns, or low-quality bases were removed. Clean reads were aligned to the reference genome (GCF_015476345.1_ZJU1.0_genomic) using the BWA software (v1.0.6) [12]. The resulting BAM files were sorted and duplicates removed using SAMtools software (v1.15.1) [13]. Subsequently, the processed BAM files underwent variant calling across multiple samples using the HaplotypeCaller module within GATK software (v4.3.0.0) [14]. Detected variants were filtered through the VariantFiltration module based on predefined criteria. Functional annotation of identified single nucleotide polymorphisms (SNPs) was conducted using ANNOVAR software (https://annovar.openbioinformatics.org/en/latest/user-guide/download/ (accessed on 1 February 2016)) [15]. To enhance the accuracy of SNP detection, additional filtering steps were applied. SNPs were filtered based on the following thresholds: QD < 2.0, FS > 60.0, MQ < 40.0, and SOR > 3.0. Stringent filtering was performed to exclude SNP clusters (no more than two SNPs within 5 bp), SNPs located within 5 bp of an indel, and closely spaced indels (no two indels within 10 bp). For any loci with a genotype quality (GQ) score below 20.0, the genotype quality of the corresponding samples was flagged as lowGQ.

### 2.3. Select Scanning Analysis

FST is a powerful tool for detecting regions under selective sweeps, particularly when analyzing functional regions closely associated with environmental adaptation, which often exhibit strong selection signals. The π ratio is calculated based on the ratio of paired intervals of population chromosomes, representing the differences in polymorphism levels between two populations. This measure reflects the extent of differential selection pressure across genomic intervals in distinct populations. DXY is a similar metric to the π ratio but focuses exclusively on inter-population comparisons, highlighting the differences in polymorphisms and indicating whether populations are subject to divergent selection pressures within the same genomic regions. To minimize the risk of false positives, we employed FST, π ratio, and DXY to analyze selection signals between the ancestral population of Lake Matahu ducks and a recently emerged white-feathered population. These analyses were conducted using the PIXY software (v1.2.7.beta1) [16], focusing on the top 5% of the genome exhibiting high differentiation under strong selective pressure. The window size for all selective sweep analyses was uniformly set to 10 kb. Genes within the regions under selection were identified for further investigation.

### 2.4. cDNA Library Construction and RNA-Sequencing

Total RNA was extracted from collected skin tissue samples, with RNA concentration and purity assessed using Nanodrop2000, RNA integrity verified by agarose gel electrophoresis, and RQN values measured using the Agilent 5300 platform.

After confirming RNA quality, samples were sent to Shanghai Meiji Bio for cDNA library construction and sequencing. The libraries were prepared using the Illumina NovaSeq Reagent Kit, and sequencing was performed on the Illumina NovaSeq X Plus platform. Post-sequencing, raw data were processed with fastp software (v0.23.2) to filter out sequencing adaptors, low-quality reads, sequences with high N content, and reads that were too short in length [17]. Clean data were aligned to the reference genome using HISAT2 software (v2.2.1) to obtain mapped data for subsequent transcript assembly and expression analysis [18]. Transcript assembly for each sample was conducted using StringTie software (v2.15.1), adopting a reference-based approach [19]. Gene and transcript expression levels were quantified using RSEM software (v1.2.27), with expression levels represented as FPKM values [20]. Differentially expressed genes (DEGs) were identified by analyzing the read counts across multiple samples using DESeq2 software (v1.44.0) [21]. Genes were considered differentially expressed if they met the criteria of false discovery rate FDR < 0.05 and |log2FC| ≥ 1.

### 2.5. Significant Difference Gene Functional Annotation and Enrichment Analysis

The annotated DEGs were uploaded to the DAVID database “https://davidbioinformatics.nih.gov/ (accessed on 11 March 2024)” for Gene Ontology (GO) and Kyoto Encyclopedia of Genes and Genomes (KEGG) enrichment analyses, with “duck” selected as the species. GO terms and KEGG pathways with a *p*-value < 0.05 were considered significantly enriched. The top 20 results from these analyses were visualized as bubble plots using the R package ggplot2 (v3.5.1).

## 3. Results

### 3.1. Genome Sequencing Results and Variant Detection Statistics

Following rigorous filtering of the sequencing data, high-quality clean reads were obtained. After removing outliers, a total of 59 samples (29 ducks in the original population and 30 in the white-feathered population) that were retained produced 2628.35 Gb of raw data, and, after filtration, 2617.1 Gb of clean data was retained, with an average of 44.36 Gb per sample. Post-filtering, the average Q20, Q30, and GC content values reached 99.08%, 96.08%, and 41.92%, respectively, all of which meet the quality control (QC) standards for subsequent analyses.

**Figure 2:**
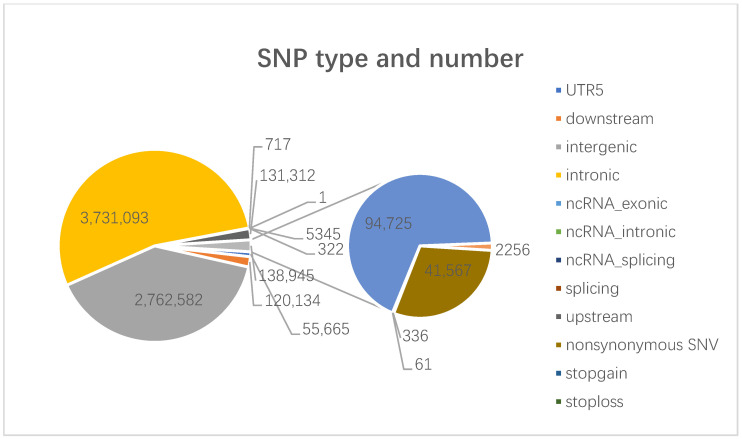
Information on the location and number of nucleotide mutation sites involved.

The filtered clean data were aligned to the reference genome, yielding an average alignment rate of 99.68% per sample and an average sequencing depth of 36.98. On average, 7,103,821 variant sites were identified per individual (Table S1), and the distribution and number of variant loci were summarized (Figure 2).

### 3.2. Analysis of Signals Selected for Feather Color Differences

**Figure 3:**
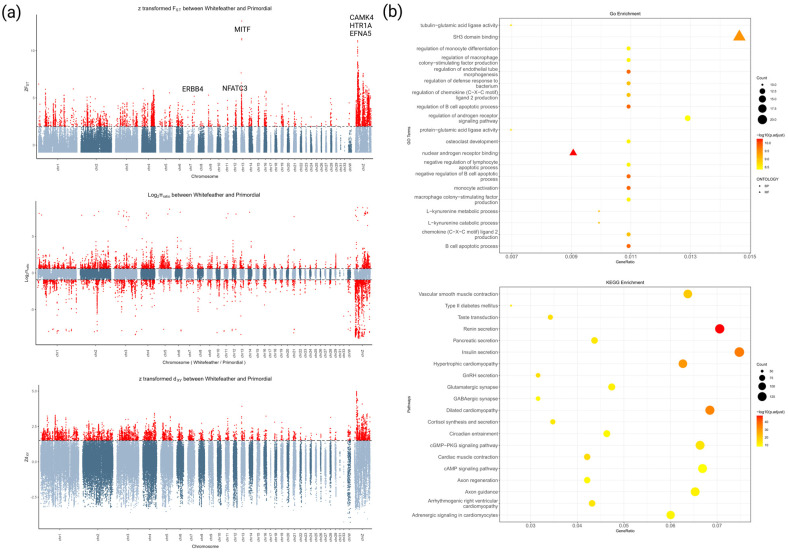
Genomic data analysis: (**a**) selection of signal analysis results; (**b**) functional enrichment of genes in selected regions.

In this study, Fst, Pi ratio, and Dxy values between the two populations were calculated separately. The top 5% of these values was used as the threshold for screening, and genes identified by at least two of the methods were designated as candidate genes. A total of 1344 candidate genes were identified, with 312 genes being detected by all three methods simultaneously (Table S2; Figure 3a).

#### GO and KEGG Annotation Analysis of Genes

To elucidate the functional characteristics of the candidate genes, we performed GO and KEGG annotation and enrichment analyses (Table S3). A total of 3512 GO terms and 323 KEGG pathways were annotated.

Of these, 794 GO terms and 70 KEGG pathways were further filtered based on a threshold of *p* < 0.05. Among the top 20 GO terms, most were associated with immune response, followed by hormone receptor regulation and protease activity. The KEGG pathways were primarily related to hormone secretion, muscle contraction, nerve development, and related processes (Figure 3b). Furthermore, L-kynurenine metabolism and catabolism, macrophage colony-stimulating factor production, retinoic acid receptor and BMP signaling regulation, neural crest cell development migration and differentiation, skin epidermal and hair follicle development, pigmentation catabolism, and several other aspects were associated with plumage color traits (Figure 3b). Similarly, KEGG pathways associated with plumage color traits were primarily linked to the cGMP-PKG, cAMP, PI3K-Akt, and MAPK signaling pathways (Figure 3b and Table 1).

### 3.3. Transcriptome Analysis of Different Tissue Parts of Matahu Ducks

The Illumina platform was utilized to construct five groups of skin transcriptome libraries (each with three biological replicates) from various populations of Matahu ducks: the original population, the newly hatched white-feathered population, the male ducks from the original population, the female ducks from the original population, and the newly hatched white-feathered population (without gender distinction). On average, each sample generated 43,663,562.27 raw reads. After removing adapter sequences and low-quality reads, an average of 43,116,877.33 clean reads per sample was obtained. The mean Q20, Q30, and GC contents of the samples were 98.43%, 95.46%, and 49.96%, respectively (Table S4). These results indicate that the 15 skin tissue transcriptome libraries constructed were of high quality and suitable for further analysis.

#### 3.3.1. Screening of Differentially Expressed Genes

In Table 2, the DEGs and their expressions status have been summarized. A total of 1406 significantly differentially expressed genes were identified, and these were treated as a candidate gene library associated with plumage color variation. These genes were then compared with the results from genome analyses, which significantly enhanced the efficiency and comprehensiveness of the study (Table S5 and Table 2).

#### 3.3.2. Gene Ontology and KEGG Annotation Analysis of Genes

**Figure 4:**
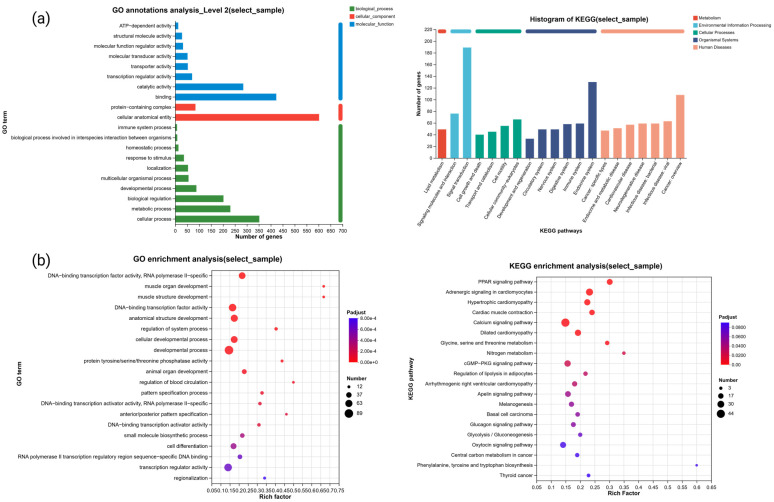
Functional enrichment of differentially expressed genes from transcriptome data: (**a**) gene annotation analysis; (**b**) gene function enrichment analysis.

The GO and KEGG functional annotation and enrichment analysis of the DEGs revealed that 1473 DEGs were mapped to 36 GO terms. The majority of these genes were annotated under the biological process category, primarily involved in cellular processes, metabolic processes, and biological regulation, among others (Table S6; Figure 4a). In the molecular function category, most genes were annotated under binding, catalytic activity, and transcription regulator activity. For cellular components, the annotations were primarily related to cellular anatomical entities.

In the KEGG pathway analysis, 2663 differentially expressed genes were annotated to a total of 323 pathways, among which the number of multiple annotated pathways, such as signal transduction, endocrine system, immune system, etc., was high.

Subsequently, a threshold of *p*-value < 0.05 was applied, and the rich factor was sorted in descending order. The top 20 results were selected for presentation (Figure 4b). The analysis revealed that the GO terms were predominantly clustered within biological processes, with significant enrichment observed in terms such as the regulation of cardioblast proliferation, the positive regulation of vasoconstriction, and the regulation of secondary heart field cardioblast proliferation. In the molecular function category, significant enrichment was found for glycogen binding, fructose 1,6-bisphosphate 1-phosphatase activity, and carbon-oxygen lyase activity acting on phosphates. For cellular components, only the troponin complex exhibited significant enrichment. These terms are primarily associated with cell proliferation, protease activity, and signal regulation.

The KEGG pathway analysis indicated significant enrichment in pathways such as phenylalanine, tyrosine, and tryptophan biosynthesis, nitrogen metabolism, and phenylalanine metabolism, which are mainly related to substance metabolism, particularly amino acid metabolism.

To identify the GO terms and KEGG pathways associated with plumage color formation, key substances and factors involved in the melanin synthesis process were used as keywords. GO terms related to plumage color were primarily associated with melanin and pigment synthesis and metabolism, pigment granules, and melanosome membranes. The relevant KEGG pathways were linked to amino acid synthesis and metabolism, the cGMP-PKG signaling pathway, and the estrogen signaling pathway (Table 3).

### 3.4. Integration of Genomic and Transcriptomic Data

**Figure 5:**
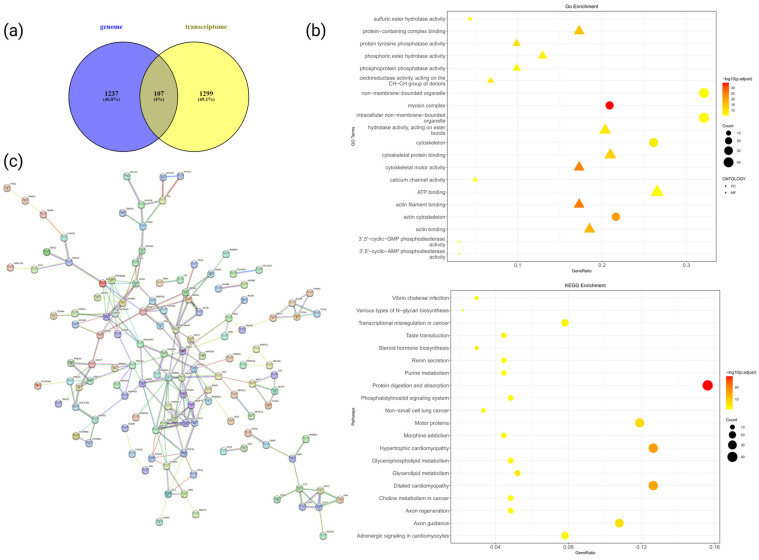
Analysis of the portion of the genome subject to selection of regions overlapping with the differentially expressed genes of the transcriptome: (**a**) Wehn diagram statistics for overlapping genes; (**b**) functional enrichment analysis of genes in overlapping sections; (**c**) analysis of protein interactions of genes and overlapping partial gene proteins involved in candidate pathways associated with plumage color traits.

To further elucidate the mechanisms underlying the formation of the white plumage trait in Lake Matahu ducks and identify the causal genes responsible for this phenotype, we conducted an integrated analysis of DEGs from transcriptome data and differentially selected genes from genomic selection sweep analysis. A total of 1344 genes were identified through the genomic selection sweep analysis, and 1473 significantly DEGs were identified from the transcriptome data, with 107 overlapping genes between the two datasets (Figure 5a). GO and KEGG enrichment analyses were performed on the overlapping genes, identifying 806 GO terms and 95 KEGG pathways. Of these, 203 GO terms and 28 KEGG pathways were significantly enriched (*p*-value < 0.05) (Figure 5b). Based on reviewing the literature, we ultimately speculated that *DGKI*, *GPRC5B*, *HMX1*, *STS*, *ADGRA1*, *PRKAR2B*, and *HOXB9* genes may play a role in plumage color traits. To explore the interactions among these candidate genes, we constructed a protein–protein interaction network using the STRING database, focusing on genes within plumage-related pathways. The analysis revealed that *FOS* proteins interact with *MITF* proteins, which may play a critical role in determining feather color phenotypes (Figure 5c).

## 4. Discussion

In the current study, we identified several key genes, such as *MITF*, *TYR*, *TYRP1*, and *MC1R*, and multiple signaling pathways, including cGMP-PKG, cAMP, and PI3K-Akt pathway, associated with melanin formation by genomic and transcriptomic analysis. We hypothesized that the expression of *DGKI*, *GPRC5B*, *HMX1*, *STS*, *ADGRA1*, *PRKAR2B*, and *HOXB9* genes is associated with feather coloration.

Consistently, it has been reported that amino acids play a critical role in melanin synthesis, particularly tyrosine, which is oxidized and subsequently absorbed by the *TYR*, leading to the formation of melanin [22]. Wang et al. identified a significant association between polymorphisms at three SNP loci of the *TYR* (c.280T>C, c.345G>A, and c.369G>A) and plumage color in domestic geese [23]. Similarly, Xu et al. reported that an increase in *TYR* expression or a decrease in *TYRP1* expression influenced the black-feather phenotype in Korean quail [24]. Wang et al. further demonstrated that the absence of both *TYR* and *TYRP1* expression in the hair follicles of Pekin ducks, compared to Liancheng white ducks, leads to insufficient melanin synthesis, which may directly cause the production of white feathers in Pekin ducks [3]. Moreover, a study proposed that *TYR* and *TYRP1* may influence the sexual dimorphism in the head plumage of mallards, possibly through the cis-regulation of transcription factors and Z-chromosome dosage effects [25].

The *MITF* is a pivotal member of the *MIT* family and acts as a central regulator of melanogenesis, controlling the expression of *TYR* and other genes critical for melanin production [26]. Mutations in *MITF* can severely impair melanocyte development, and the protein is essential for the survival of both embryonic and adult melanocytes. Furthermore, *MITF* is considered a key regulator of melanocyte differentiation and proliferation [27]. Two synonymous SNPs (c.114T>G and c.147T>C) and a 14 bp indel (GCTGCAAAC AGATG) in intron 7 of the *MITF* gene have been significantly associated with black and white-feather varieties [28]. Lin et al. found eight loci in the *MITF* promoter that were significantly associated with black and white-feather changes [29]. Genome-wide association studies in Mallard ducks, Pekin ducks, and other local breeds revealed that the *MITF* gene is the principal determinant of white feathers, controlling both melanin deposition and melanocyte proliferation. A 6.6 kb insertion between exon 1 M and exon 2 of *MITF* was shown to alter spliceosome transcript levels, leading to the albino plumage phenotype observed in Pekin ducks [30]. Additionally, the hypermethylation of the *MITF* promoter region was associated with decreased gene expression in white quail, correlating with the white-feather phenotype [31].

The melanocortin receptor family, particularly *MC1R* and *MC5R*, also plays a crucial role in skin pigmentation. The *MC1R* encodes a G protein-coupled receptor that mediates pigmentation through the binding of ligands such as α-melanocyte-stimulating hormone (α-MSH) and adrenocorticotropic hormone (ACTH). Upon ligand binding, *MC1R* activates receptor-coupled G proteins on melanocyte membranes, leading to the conversion of adenosine triphosphate (ATP) into cyclic AMP (cAMP), which subsequently activates *TYR* and stimulates melanin production [32]. Fan et al. identified a 100% correlation between the g.18838624 T>C variant in *MC1R* and feather color in Taiwanese chickens, with the CC (E1) genotype associated with MTH and the TT (E2) genotype linked to HTH [33]. Additionally, Liu et al. reported a significant differential expression of *MC1R* in the plumage stems of Black Woolly Ducks and GF2 Ducks, suggesting that *MC1R* may be a key regulator of melanosis, including traits such as spotty black feathering. They identified several regulatory loci that modulate *MC1R* expression [34]. Functional studies on the Opioid Receptor Mu 1 (*OPRM1*) have primarily focused on neuronal regulation, but its role in skin pigmentation remains unclear. However, it is hypothesized that *OPRM1* may influence skin color due to the shared embryonic origin of the skin and nervous system. Additionally, *OPRM1* is expressed in both keratinocytes and melanocytes, where it may contribute to cell differentiation [35]. Finally, the ATP Binding Cassette Subfamily B Member 6 (*ABCB6*), a multifunctional transporter protein, is highly expressed in melanocytes, suggesting its involvement in transporting key enzymes and proteins necessary for melanin synthesis. Recent studies have demonstrated that *ABCB6* is a critical regulator of melanogenesis via the GSK3-β/β-catenin signaling pathway [36].

Melanin synthesis involves numerous regulatory factors, and, as a result, plumage color variation is influenced by multiple signaling pathways. In this study, several important pathways, including cGMP-PKG, cAMP, PI3K-Akt, MAPK, and BMP, were found to be closely associated with melanin formation [37,38,39,40]. Additionally, processes such as amino acid synthesis and metabolism, the dynamic development and differentiation of neural crest cells, and the growth and development of skin and hair follicles are essential for both plumage pigmentation and overall development [41,42]. Several genes, including Nuclear Factor of Activated T Cells 3 (*NFATC3*), Calcium/Calmodulin-Dependent Protein Kinase IV (*CAMK4*), 5-hydroxytryptamine Receptor 1A (*HTR1A*), Ephrin-A5 (*EFNA5*), and Protein Kinase AMP-Activated Non-Catalytic Subunit Gamma 1 (*PRKG1*), have been shown to influence melanocyte proliferation, tyrosinase activity, and melanin synthesis through various signaling pathways, such as harmine/DYRK1A, CaMK4-p-CREB, MAPK, and nitric oxide/cGMP [43,44,45,46,47]. For example, in cattle, *EFNA5* is associated with white frontal stripes [48], and Heart and Neural Crest Derivatives Expressed 2 (*HAND2*) is differentially expressed in the fin pigmentation of cichlid fishes and in subspecies of the black-eyed cuckoo, affecting pigmentation in these species [49,50]. Furthermore, genes such as Heat Shock Protein Family A (Hsp70) Member 8 (*HSPA8*), Erb-B2 Receptor Tyrosine Kinase 4 (*ERBB4*), Estrogen Receptor 1 (*ESR1*), and HRas proto-oncogene, also known as GTPase (*HRAS*) play crucial roles in melanoma transformation, impacting melanogenesis, metastasis, melanocyte division, and apoptosis. These genes are also considered biomarkers for melanoma diagnosis [51,52,53,54]. The *HSPA8* gene, in particular, has been shown to be upregulated in response to cold stimulation in both carp and zebrafish, indicating a potential role in pigmentation regulation under environmental stress conditions [55,56].

Despite these advancements, relatively few studies have explored the impact of genes such as Diacylglycerol Kinase Iota (*DGKI*), G Protein-Coupled Receptor Class C Group 5 Member B (*GPRC5B*), H6 Family Homeobox 1 (*HMX1*), Steroid Sulfatase (*STS*), Adhesion G Protein-Coupled Receptor A1 (*ADGRA1*), Protein Kinase CAMP-Dependent Type II Regulatory Subunit Beta (*PRKAR2B*), and Homeobox B9 (*HOXB9*) on plumage color. The specific mutations and functions of these genes require further investigation. In this study, we provide theoretical insights into how these genes may influence plumage color traits, laying the groundwork for future molecular validation experiments. For instance, diacylglycerol (DAG) has been shown to increase melanin content in human melanocytes in vitro and enhance pigmentation in guinea pigs in vivo [57,58]. *DGKI*, an enzyme involved in the phosphorylation of DAG, regulates intracellular signaling and metabolic processes. Kawaguchi et al. reported a significant correlation between *DGK* gene expression, melanin content, tyrosinase activity, and tyrosinase protein levels [59]. The PKA signaling pathway also plays an important role in regulating melanogenesis, with *PRKAR2B*, a regulatory subunit, potentially influencing melanogenesis by modulating PKA pathway activity [60,61]. *STS*, an enzyme involved in the metabolism of estrogen precursors, enhances tyrosinase activity, which leads to increased melanin synthesis in extracellular melanocytes when physiological estrogen levels are elevated [62]. Moreover, *HOXB9* and *HMX1* play critical roles in embryonic development, tissue morphogenesis, and neural development. The *HOXB9* is a target of the Wnt/β-catenin signaling pathway, which is crucial for melanocyte development and differentiation [63]. Transcriptome profiling has revealed differential expression of *HOXB9* in black and white-feather bulbs of black-boned chickens [64]. Furthermore, it has been suggested that melanocyte development from neural crest cells shares signaling molecules with chevron cells, and the elimination of *HMX1* expression through RNA interference leads to a near-complete loss of neurogenesis, with SOX10 cells around the dorsal root ganglion (DRG) acquiring *MITF* expression [65]. *FOS*, an immediate early gene (IEG), is rapidly induced following cellular stimulation and triggers programmed cell death (PCD). Wang et al. found that in rats and humans, *FOS* functions as a transcription factor regulating Becn1/BECN1 transcription and promotes cellular autophagy, although the corresponding regulatory site in mice is absent [66]. Furthermore, a study hypothesized that in mouse cells, *FOS* upregulates *BECN1* expression through CREB phosphorylation, similar to the c-Fos/CREB cycle in engram cells, and induces autophagy downstream [67]. Furthermore, *STAT3* and *MITF* synergistically induce cellular transformation by upregulating c-Fos expression [68]. While *MITF* typically binds to high-affinity E/M-box motifs, when *MITF* expression is high, downregulated genes tend to contain FOS/JUN/AP1/ATF3 loci [69].

It is interesting to note that the significant difference in plumage color between male and female ducks suggests that sex-linked genes may be involved in the regulation of this trait, and this is indirectly supported by the fact that a significant portion of the genes in the data results are located on the sex chromosomes. We know that the genes on the sex chromosomes not only affect the sex but also may affect the expression of autosomal genes related to feather traits, and the regulation process is complex. However, our study lacks experimental validation to confirm this hypothesis. Thus, we recommend that future studies with a focus on the experimental characterization of sex-linked and autosomal genes could provide a clearer understanding of the genetic mechanisms of feather variation.

## 5. Conclusions

This study employed genomic selection elimination analysis and transcriptomic differential expression analysis to investigate genomic variants associated with plumage traits across different plumage groups and body regions of Matahu ducks. Several candidate genes and signaling pathways related to plumage color variation were identified, including *MC1R*, *TYR*, *TYRP1*, *ABCB6*, *BLVRA*, and *MC5R.* Furthermore, the expression of *DGKI*, *GPRC5B*, *HMX1*, *STS*, *ADGRA1*, *PRKAR2B*, and *HOXB9* genes was found to potentially play a significant role in plumage color traits. These findings provide a valuable research foundation for the further exploration of the genetic mechanisms underlying plumage color variation in ducks.

## Figures and Tables

**Table 1:** Genes and their regulated signaling pathways associated with plumage features.

ID	Description	*p*-Value	Genes
ko04022	cGMP-PKG signaling pathway	1.89 × 10^−16^	*NFATC3*
ko04024	cAMP signaling pathway	3.99 × 10^−10^	*CAMK4*, *HTR1A*
ko04151	PI3K-Akt signaling pathway	1.53 × 10^−9^	*EFNA5*, *ERBB4*
ko04010	MAPK signaling pathway	0.0001860	*EFNA5*, *NFATC3*, *ERBB4*
ko05218	Melanoma	0.0414223	*MITF*

**Table 2:** Summary of DEGs identified in different subgroups.

Group	Total DEG	Up	Down
YGB_vs_BYB	82	71	11
YMB_vs_BYB	101	76	25
YGB_vs_YMB	315	137	178
YGB_vs_YGN	843	202	641
YGB_vs_YGF	52	38	14
YGN_vs_YGF	832	609	223

Note: YGB: dorsal skin of original group male ducks; YGN: neck skin of original group male ducks; YGF: abdominal skin of original group male ducks; YMB: dorsal skin of original group female ducks; BYB: dorsal skin of white-feathered ducks (sex not differentiated).

**Table 3:** DEGs and their regulated signaling pathways associated with plumage features.

GO ID	Description	*p* Value	Gene_Name
GO:0004977	Melanocortin receptor activity	0.0082759	*MC1R*, *MC5R*
GO:0001755	Neural crest cell migration	0.0082356	*HAND2*
GO:0042440	Pigment metabolic process	0.0082356	*MC1R*, *TYR*, *TYRP1*
GO:0006582	Melanin metabolic process	0.0082759	*MC1R*, *TYR*, *TYRP1*
GO:0042438	Melanin biosynthetic process	0.0082759	*MC1R*, *TYR*, *TYRP1*
GO:0090741	Pigment granule membrane	0.0136354	*ABCB6*, *TYR*, *TYRP1*
GO:0033162	Melanosome membrane	0.0136354	*ABCB6*, *TYR*, *TYRP1*
GO:0046148	Pigment biosynthetic process	0.0290307	*MC1R*, *TYR*, *TYRP1*
map00260	Glycine, serine and threonine metabolism	4.93 × 10^−5^	*GATM*, *GAMT*
map04022	cGMP-PKG signaling pathway	5.54 × 10^−4^	*ATP2A2*, *PRKG1*
map04915	Estrogen signaling pathway	4.10 × 10^−2^	*HRAS*, *HSPA8*, *ESR1*

## Data Availability

The datasets analyzed in this study are available from the corresponding author upon reasonable request.

## Supplementary Materials

The following supporting information can be downloaded at https://www.mdpi.com/article/10.3390/ani14213111/s1: Table S1: Genome Sequencing Data Statistics; Table S2: Analysis results of three selective signal methods; Table S3: Functional enrichment analysis of candidate genes in selected regions; Table S4: Transcriptome sequencing result statistics; Table S5: Summary of differentially expressed genes in different subgroups; Table S6: Functional enrichment analysis of differentially expressed genes.
